# Anti-pulling force and displacement deformation analysis of the anchor pulling system of the new debris flow grille dam

**DOI:** 10.1038/s41598-022-07722-2

**Published:** 2022-03-08

**Authors:** Yongsheng Wang, Baohong Lv, Jianshe Liu, Xiaobin Zhang

**Affiliations:** 1 School of Civil Engineering, Lanzhou University of Technology, Lanzhou, 730050 China; 2 Key Laboratory of Disaster Prevention and Mitigation in Civil Engineering of Gansu Province, Lanzhou University of Technology, Lanzhou, 730050 Gansu China

**Keywords:** Ecology, Natural hazards

## Abstract

To avoid waste from a large section space structure layout and deep burial, improve the structural strength and stability. Anchor technology is introduced, and combined with the advantages of the supporting wall, a new debris-flow grille dam is proposed. Starting from the force process and damage mechanism of the new debris-flow grille dam, the computation formula for the anti-pulling force and the total displacement is given. The anti-pulling force includes the sidewall frictional resistance of the anchor pier and the positive pressure of the front end face of the anchor pier. The total displacement includes three parts: the elastic deformation of the cable, the relative shear displacement between the anchor pier and the surrounding soil, and the compression deformation of the soil at the front of the anchor pier. Finally, the influence of soil parameters and anchor pier size on the anti-pulling force and displacement deformation of the anchor-pulling system is analyzed by examples, and the results are compared with the numerical results. The results show that the displacement deformation decreases gradually with increasing elastic modulus of the soil around the anchor pier and increases with increasing Poisson's ratio. The change in elastic modulus mainly affects the relative shear displacement of the anchor pier and soil and the compressive deformation of the soil at the front end of the anchor pier. Poisson's ratio has the greatest influence on the relative shear displacement of the anchor pier and soil. A larger anchor pier is not better; thus, it is wise to choose the economic design dimension. Theoretical and numerical simulation results are consistent, showing a linear growth trend. The results of this paper can further improve the theoretical calculation method of the new debris-flow grille dam, thus making it widely used in more debris flow control projects.

## Introduction

The anchoring technique or anchor method, the general designation of design and construction technology. It refers to the skill that one end of the tension component is fixed to the rock and soil body and the other is linked with engineering structures. To with stand the thrust or the uplift force originated from the earth pressure, water pressure or other external force applied to the structure using the internal resistance of rock and soil body can be utilized^1^. Commonly used anchorages in engineering include rods using rigid components as pull rods and anchor cables using flexible components as pull rods, which are collectively called anchors^2,3^.

The earliest use of anchoring technology can be traced back to the nineteenth century. In 1872, the anchor reinforcement slope was first used in open shale mines in North Wales in British; in 1912, the bolt support underground mine roadway was first used in the Seleznev mine in Germany; in 1915–1920, bolt support technology was gradually used in metal mines in the US; before 1924, anchor spray support was used in the Donbass mine in the Soviet Union^4,5^. In China, over the past decade, the rock-soil anchoring technique has thrived in the field of rock and soil reinforcement because of its unique effect, simple process, wide range of uses and economic cost. Anchoring technology has touched almost every corner of the field of civil engineering, such as mine shafts, railway tunnels, underground chamber supports, geotechnical slope reinforcement, foundation stability, deep foundation pit support, anti-floating and anti-overturning structures, underground tensile structures of suspension-cable buildings, construction, reconstruction and expansion of concrete structures. The progress of anchoring technology will effectively promote the development of civil engineering industries and promises a very broad application^6,7^.

Ground anchors, as an anchoring technique, are widely used in the construction of towers and cables^8^. It is also commonly used to anchor and tract the wire rope, brake the wire rope, winch, cable and other load-carrying equipment, and pass an external force to the foundation. Ground anchors are an important load-carrying part that is directly related to the safety of tower construction, economy and rationality^9,10^. The effectiveness of ground anchoring mainly depends on the interaction of soil and ground anchors, and its mechanical mechanism of load transfer is often originated from a potential displacement of the anchor or soil^11^. Accordingly, ground anchors can be divided into two types: active and passive. Active ground anchors, which load onto the anchor initiatively, keep the soil relatively motionless. Therefore, the interaction of anchors and soil is originated from the stretching and displacement of the anchor. The anchor used to support the superstructure load is one particular instance, and the pull-out test is the same. On the other hand, passive ground anchors, which anchor into the soil, constrain the potential displacement of the soil. Therefore, the interaction of anchors and soil is mainly originated from soil displacement. Tunnel support structures, retaining walls and soil displacement control of slope stability are examples of such applications of anchors^12^. the first real non-destructive testing method that used Schmidt hammer rebound number to estimate the load carrying capacity of anchor bolts was successfully proposed by the authors. In this method the authors successfully relate the pull-out strength of the steel anchors, N, embedded in the concrete with the Schmidt hammer rebound number, R. The research team was successful in identifying anchor bolts with improper installation resulting in lower pull-out strength^13^.

In the prevention and control engineering of debris flow, to resist the impact of debris flow fluid and large stones, a large section of structural component is often designed to enhance the lateral stiffness of grille dam, deep-bury about half the length of the pile and arrangement forms of spatial structure are designed to guarantee the stability of the grille dam^14–16^. This design concept is feasible for a single dam when a large-scale debris flow occurs but is very wasteful for medium- and small-scale debris flow^17^ and integrated control of the entire river basin^18–21^. In this paper, the ground anchor technology is firstly introduced into the new type of grid dam structure of debris flow control project. This technique skillfully combines the advantages of ground anchors and buttresses to ensure not only the stiffness of the dam body, but also the reliability and stability of the dam body^22^.

Turkyilmazoglu M developed a valid analytical formula unifying all the well-documented results into a single one. The most generic data from the obtained wave run-up formula can be combined to study the many coasts of convex/concave bottoms in the world. The results verify that shores with a convex bottom profile has higher maximum wave run-up than that corresponding to a concave bottom profile^23^. To improve the theoretical calculation method of the new debris flow grille-dam structure and make it widely used in more areas and fields, the paper starts from the loading process and failure mechanism of the new debris flow grille-dam system and then analyzes the anti-pulling force and displacement deformation of the anchor-pulling system. The anti-pulling force mainly consists of the frictional resistance of the lateral walls of the anchor piers and the positive pressure on the front of them; the total displacement is comprised of the elastic deformation of the stayed cable, the relative shear displacement originated from the anchor pier and surrounding soil and the compression deformation of the soil in front of the anchor pier. Finally, the author tests and verifies the correctness of the calculation model through numerical simulation and analyses the influence of the thickness of the overlying soil, the shapes of the anchor pier and other factors on the anti-pulling force and displacement deformation of the anchor-pulling system.

## The new debris flow Grille-dam

### Introduction of the new debris flow Grille-dam

**Figure 1:**
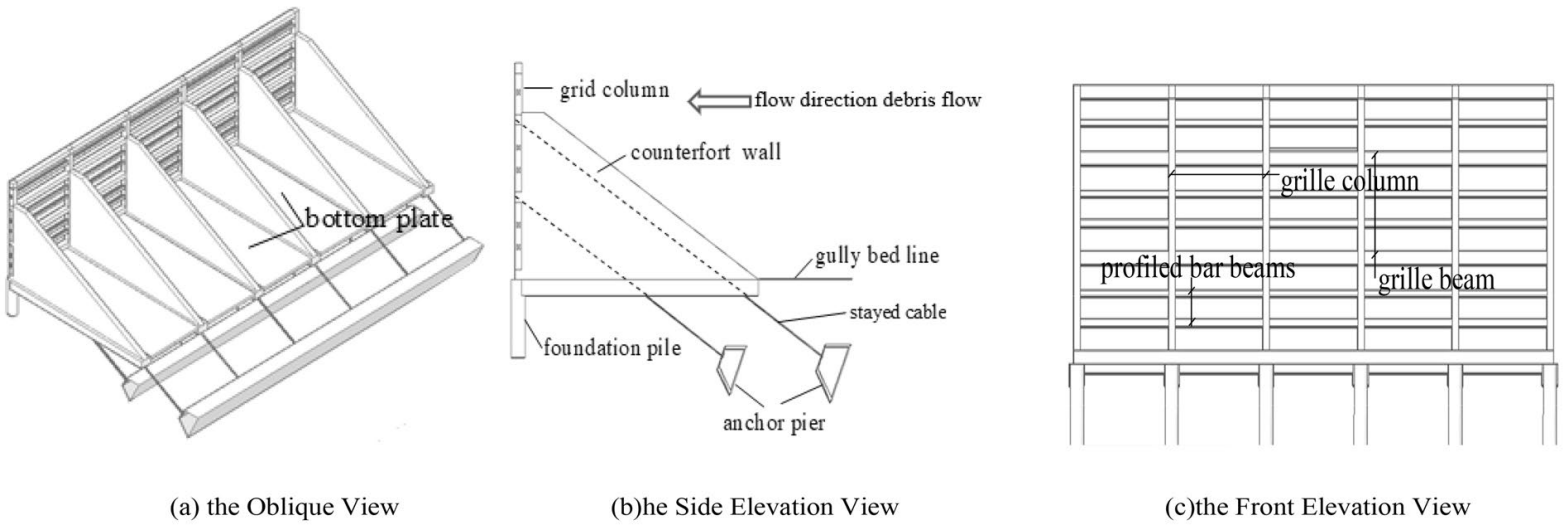
The structure for the Grille-dam.

The new debris-flow grille dam is mainly composed of grilles, which consist of columns, beams and waste profiled bar beams, foundation piles, counterfort walls, stayed cables, anchor piers, bottom plates and bottom beams. As shown in Fig. 1. Grillage Dam is formed by the combination of reinforced concrete column, beam and section steel beam. Moreover, the stayed cable of the ground anchor can guarantee the integral stability of this new grille dam. In addition, the reinforced concrete counterfort walls play a role in protecting the stayed cable from being broken and in splitting the flow, which makes the stress on the grille dam relatively even.

### Features

The new structure is a kind of permeable structure with strong stability, good permeability and less material consumption for debris flow control. Its main characteristics are: leaving larger gravel, releasing the lower harm to the solid particles, realize the block-row combination; controlling the diameter of the releasing solid particles by adjusting the spacing of the grille; keeping the fluvial equilibrium by adjusting the proportion and composition of retaining and draining; improving the structure’s service life by adjusting the gate hole to make the fine-grained materials in the pool washed away by using the running water after rainy season; draining water rapidly to reduce water pressure and uplift pressure the structure withstanding and to improve the stability of the structure; conducive to prefabricated and light development, saving material and shortening construction period.

### Scope of application

According to the experience of disaster site research and the success or failure of existing control structure^24^, the new debris-flow grille dam is mainly suitable for controlling middle- and small-scale debris flows. It is also suitable if there are fewer large stones and large floating rocks in the material source or if the channel fall is relatively low and flat. It can be used as a single structure in controlling debris flows or combined with other structures in the comprehensive control of the whole basin.

## Calculation model of the anti-pulling force for the anchor-pulling system

### The failure mechanism of anchor-pulling system

#### Mechanical composition

When the new grille dam is under the pressure of debris flow, pressure of debris flow deposits, hydrodynamic pressure and impact force of crossing the dam debris flows. The force on the dam body is transmitted to the stayed cable through the grille columns and beam nodes and then to the anchor piers in the deep stable region, eventually realizing the requirements of the safety and stability of the dam body and putting the structure under reasonable load. The anti-pulling force of the anchor-pulling system is comprised of two parts, the frictional resistance between the lateral walls of the anchor piers and the soil body as well as the positive pressure on the front of the anchor piers, which belongs to the frictional-end pressure anchor-pulling system.

#### Failure modes

Turkyilmazoglu M devoted to derive an analytical solution to the air blast response of dynamic compaction process of a sandwich composite containing a deformable front face and a cellular core. Shock wave model in association with the mass conservation and Newton’s second law are employed to solve the problem. Both the weak fluid–structure interactions (FSI) such as those that are thought to occur due to air blast loading and the non-FSI cases are discussed and the results are compared with those available in the literature^25^. The failure of the anchor-pulling system mainly takes three forms: the failure of the stayed cable, the failure of the connection with anchor heads and anchor piers, and the failure of the soil body around the anchor piers.

The failure of the stayed cable: the anchor piers deeply buried in the stable region can provide a huge anchoring force. The strength of the stayed cable becomes a weak link that the steel strand may be fractured and reinforcement yielding may occur under the great impact of debris flows.

The failure of connections with anchor heads and anchor piers: theoretically, when the strength of the stayed cable is great enough, the connections between the stayed cable and anchor heads related to the grille beam nodes or the connections between the stayed cable and anchor piers become the weakest link. If there are construction quality problems on the joint or the connection is impacted by large stones in the debris flows, the stayed cable will be pulled out of anchor piers or grille beam nodes, resulting in the failure of connections.

The failure of the soil body around the anchor piers: When the strength of the stayed cable is great enough and the connections between the stayed cable and anchor piers or grille beam nodes are reliable enough, the failure of the soil body around the anchor piers will take place.

Among the three forms mentioned above, the first two forms can easily be prevented by selecting reasonable structural material parameters and setting a variety of structural measures. This paper only discusses the third failure of the soil body around anchor piers. Now, we take an equivalent anchor-pulling system as the object to discuss. The failure of the soil body around the anchor piers may be classified into 4 phases.

**Figure 2:**
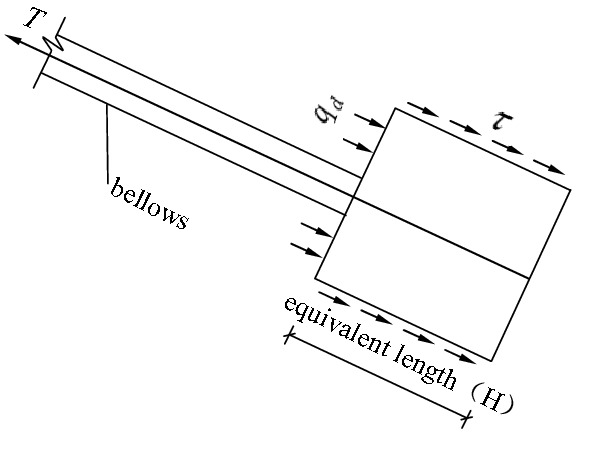
The force diagram of the anchor-pulling system in the phase of earth pressure at rest.

The first phase: The phase of earth pressure at rest. As shown in Fig. 2. In the figure, ${q}_{d}$ is the positive pressure strength of soil acting on the front face of anchor pier; $\tau$ is the shear stress on the surface of the equivalent anchor pier. When the external load on the stayed cable is relatively small, the lateral walls of the anchor piers bear frictional resistance, while the front of them bear earth pressure at rest. In this phase, the displacement of the anchor-pulling system is small, and the force–deformation properties of the system are determined by the distortion of the stayed cable.

**Figure 3:**
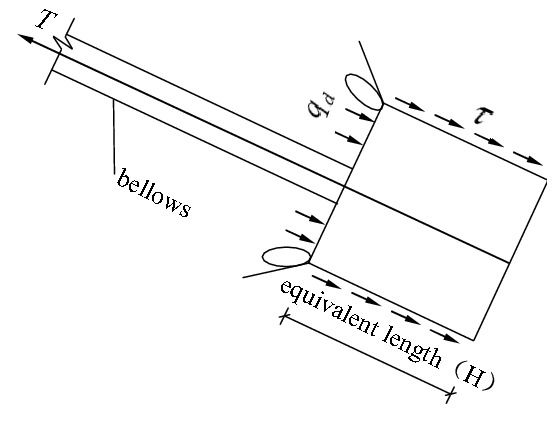
Force diagram of the anchor-pulling system in the phase of transition (emergence of plastic zone).

**Figure 4:**
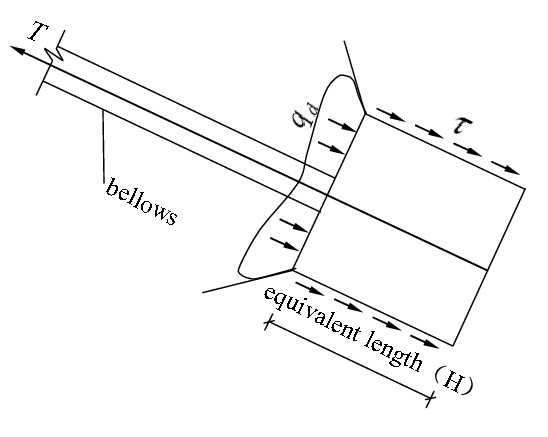
Force diagram of the anchor-pulling system in the phase of transition (joint of plastic zone).

The second phase: The phase of transition. After the frictional resistance of the lateral walls of the anchor piers peaked, if the external tension on the stayed cable continued to grow, the anchor piers began to move forward, the pressure on the front of them increased, and the soil body in the front began to produce a local plastic zone, as shown in Fig. 3. If the external tension on the stayed cable continues to grow, the plastic zones of the soil body will expand and finally be jointed to form a whole, as shown in Fig. 4. Before this phase, the force–deformation properties of the system are determined by the frictional resistance of the anchor piers. Afterwards, it is determined by the compression performance of the soil on the front of anchor piers. As the compression deformation of the soil is greater than the frictional deformation, one of the significant features is that an inflection point appears on the tension-displacement curve. After it, the slope of the curve decreases, and the displacement increases. This inflection point can be referred to as “the end pressure inflection point”.

**Figure 5:**
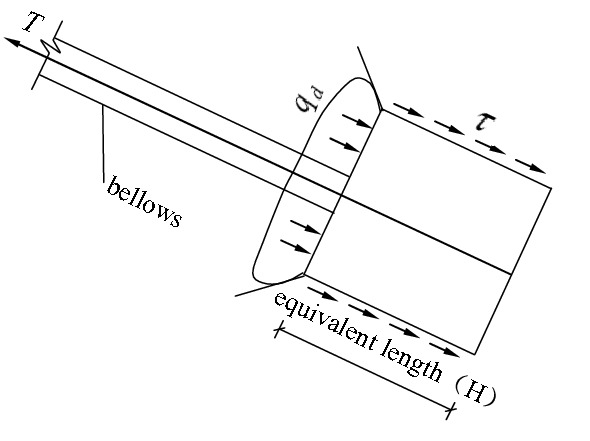
Force diagram of the anchor-pulling system in the phase of compaction-expansion in the plastic zone (expansion of plastic zone).

**Figure 6:**
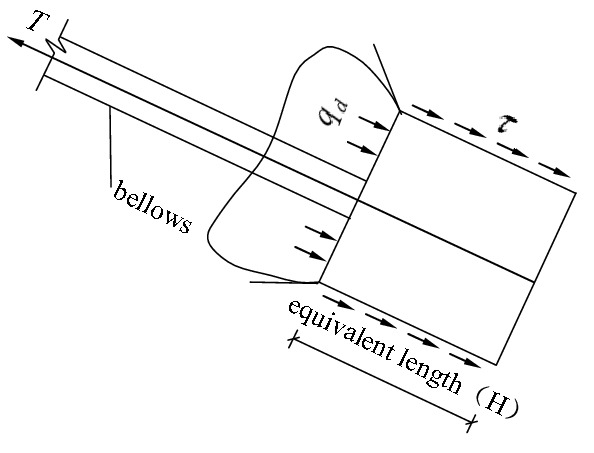
Force diagram of the anchor-pulling system in the phase of compaction-expansion in the plastic zone (stability of compaction).

The third phase: The phase of compaction-expansion in the plastic zone. After the end pressure inflection point, if the external tension on the stayed cable keeps growing, the anchor piers will have large displacement forward, and under the constraint of confining pressure of the surrounding soil along with the pressure of anchor piers, compression and stress redistribution of the soil body in the plastic zone will occur, as shown in Fig. 5. When the embedded depth of anchor piers is relatively large, as the external tension on the stayed cable grows. As the soil is continuously compacted, the resistance provided by the compacted soil to the anchor pier increases, and the displacement of the pull-anchor system tends to converge and stabilize,/n/n, as shown in Fig. 6.

**Figure 7:**
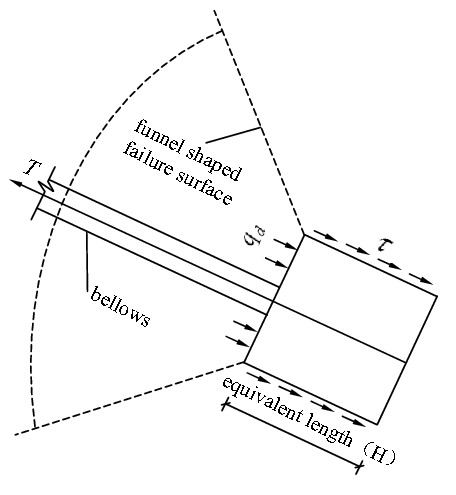
The force diagram of the anchor-pulling system in the phase of the anchor piers being pulled out.

The fourth phase: The phase of the anchor piers being pulled out. When the compaction in the plastic zone of the soil on the front of the anchor piers is stable, with the further increase in the external tension on the stayed cable, the deadweight of the overlying soil of the anchor piers cannot balance the oblique pulling force of the stayed cable and finally tends toward the limiting equilibrium condition. If the tension of the external load on the stayed cable increases to be large enough, the soil on the front of the anchor piers will form a "funnel-shaped" failure surface, and the anchor piers with the surrounding soil will be pulled out, as shown in Fig. 7.

### Calculation model of the anti-pulling force for the anchor-pulling system

**Figure 8:**
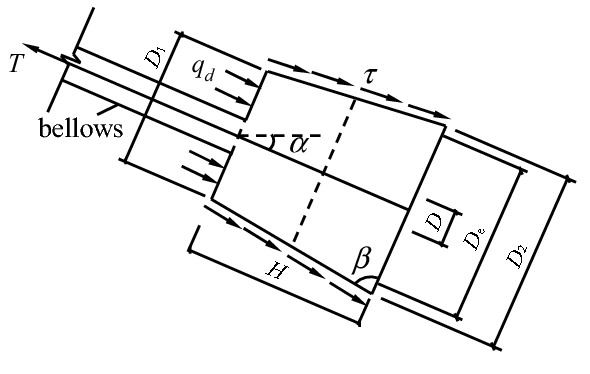
The mechanical model of trapezoid anchor piers.

**Figure 9:**
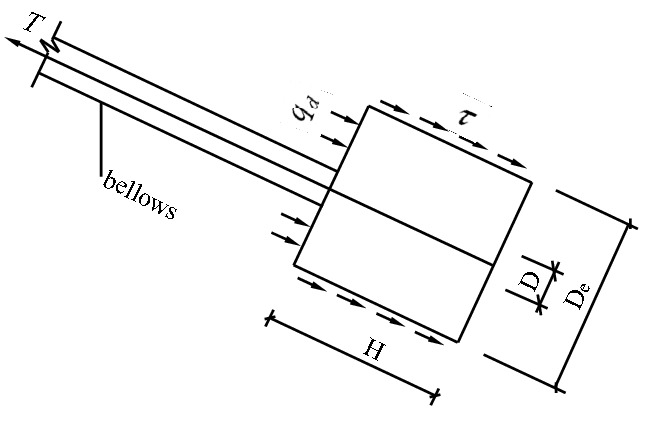
The mechanical model of equivalent rectangular anchor piers.

Considering the third form of failure above, the mechanical model is shown in Fig. 8 and Fig. 9. For the convenience of formula derivation and engineering applications, the trapezoid anchor piers in Fig. 8 can be replaced by the equivalent rectangular anchor piers in Fig. 9. In the figure, $T$ is the pulling force of anchor system.

The anti-pulling force of the anchor-pulling system $$ T_{b}$$ is composed of two parts: the anti-pulling force provided by the frictional resistance of the lateral walls $$T_{1}$$ and the soil positive pressure on the front of the anchor piers $$T_{2}$$. That is:

1 $$ T_{b} = T_{1} + T_{2} $$

where $T_{1}$ is the anti-pulling force provided by the frictional resistance of the lateral walls and can be calculated using the following equation:

2 $$ T_{1} = 2\left( {D_{e} + L_{m} } \right) \cdot H \cdot \tau $$

$T_{2}$ is the soil positive pressure on the front of the anchor piers and can be calculated using the following equation:

3 $$ T_{2} = \left( {D_{e} L_{m} + \frac{\pi }{4}D^{2} } \right)q_{d} $$

Here, $D_{e}$ is the section width of the equivalent anchor piers, $D_{e} = \left( {D_{1} + D_{2} } \right)/2$; $L_{m}$ is the section length of the equivalent anchor piers; $H$ is the section height of the equivalent anchor piers; $\tau$ is the intensity of frictional resistance between the lateral walls of the anchor piers and the formation^26^ (earth fill: 9–13 kPa; silt: 20–40 kPa; pebble: 55–65 kPa); $D$ is the diameter of the corrugated sleeve of the protective stayed cable; and $q_{d}$ is the intensity of the soil’s positive pressure on the front of the anchor piers.

## Calculation model of deformation for anchor-pulling system

When the debris flow load is applied to the new grid dam structure, the load is transferred to the deep anchor system along the beam-column junction of the dam body. When the pull force is greater than its ultimate tensile strength; When the lateral friction resistance of anchor pier is greater than the ultimate shear strength of soil or the soil at the front of anchor pier is compressed; Plastic zone development through. The structure will break down.. Therefore, the total displacement of the system $$\left( S \right)$$ can be expressed using elastic deformation of the stayed cable $$ (S_{1} )$$, relative shear displacement between anchor piers and the surrounding soil $$(S_{2} )$$ and the compression performance of the soil on the front of anchor piers $$\left( {S_{3} } \right)$$, that is, $$\left( S \right) = (S_{1} ) + (S_{2} ) + (S_{3} )$$.

### Analysis of elastic deformation of the stayed cable

As the pulling force of the stayed cable is the external load transmitted by the grille columns and beam nodes, according to Hooke's law, we know that:

4 $$ \left( {S_{1} } \right) = \frac{{TL_{l} }}{{E_{l} A_{l} }} $$

where $L_{l}$ is the length of the stayed cable; $T$ is the pulling force that the stayed cable bears; $E_{l}$ is the elastic modulus of the stayed cable; and $A_{l}$ is the sectional area of the stayed cable.

### Analysis of the relative shear displacement between anchor piers and the surrounding soil

**Figure 10:**
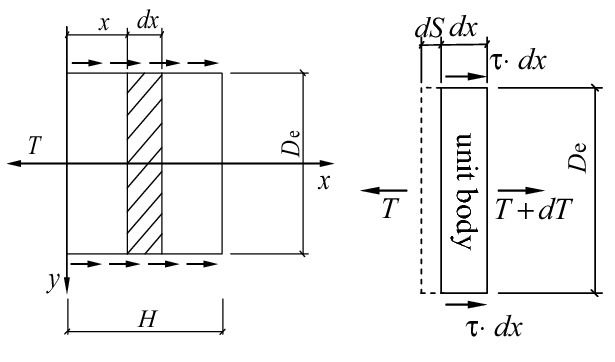
Analytical diagram of the load transfer of the anchor piers.

To analyze the relative shear displacement between equivalent anchor piers and the surrounding soil, we take anchor piers as free bodies and take one of them as a unit body. Its force is shown in Fig. 10.

From the static equilibrium of the unit body, there exists:

5 $$ \frac{\partial \sigma }{{\partial x}} = - \frac{{2\left( {L_{m} + D_{e} } \right)\tau }}{{A_{e} }} $$

where $\sigma$ is the section mean normal stress of the equivalent anchor piers, $\sigma = T/A_{e}$; $\tau$ is the intensity of frictional resistance between the lateral walls of the anchor piers and the formation; $A_{e}$ is the section area of the equivalent anchor piers, $A_{e} = D_{e} L_{m}$; and $D_{e}$ is the section width of the equivalent anchor piers.

So axial strain of unit body turns to:

6 $$ \varepsilon = \frac{\partial S}{{\partial x}} = - \frac{\sigma }{{E_{e} }} = - \frac{T}{{D_{e} L_{m} E_{e} }} $$

where $\left( S \right)$ is the displacement of the unit body; $L_{m}$ is the section length of the equivalent anchor piers; and $E_{e}$ is the elastic modulus of the equivalent anchor piers.

If we assume that there is a linear elastic incremental relationship^27^ between the shear force and shear displacement of the soil around the anchor piers, then

7 $$ 2\left( {L_{m} + D_{e} } \right)\tau = G_{s} S $$

where $G_{s}$ is the shear modulus between the anchor piers and soil interfaces, the physical meaning of which is that the shear force is produced by unit shear displacement on the unit section length of the equivalent anchor piers. After differentiating Eq. (6) and solving Eqs. (5) and (7) simultaneously, we can obtain the second-order differential equation of load transfer of the anchor piers.

8 $$ \frac{{\partial^{2} S}}{{\partial x^{2} }} - \frac{{G_{s} S}}{{D_{e} L_{m} E_{e} }} = 0 $$

Let $G_{s} a = \sqrt {G_{s} /D_{e} L_{m} E_{e} }$, the general solution of the equation above is:

9 $$ S\left( x \right) = C_{1} exp\left( {ax} \right) + C_{2} exp\left( { - ax} \right) $$

Substituting the boundary conditions of $\left( {\frac{\partial S}{{\partial x}}} \right)_{x = 0} = - \frac{T}{{D_{e} L_{m} E_{e} }}$ and $\left( {\frac{\partial S}{{\partial x}}} \right)_{H = 0} = 0$, the relative shear displacement of the anchor piers is obtained.

10 $$ S\left( x \right) = \frac{{T\left[ {exp\left( a \right) + exp\left( {2aH - ax} \right)} \right]}}{{aD_{e} L_{m} E_{e} \left[ {exp\left( {2aH} \right) - 1} \right]}} $$

Substituting $x = 0$ and $x = H$ into the equation above, we can obtain the relative shear displacement of the top and bottom of the anchor piers:

11 $$ \begin{aligned} & S\left( 0 \right) = \frac{T}{{aD_{e} L_{m} E_{e} }} \cdot \frac{{exp\left( {2aH} \right) + 1}}{{exp\left( {2aH} \right) - 1}} \\ & S\left( H \right) = \frac{T}{{aD_{e} L_{m} E_{e} }} \cdot \frac{{2exp\left( {aH} \right)}}{{exp\left( {2aH} \right) - 1}} \\ \end{aligned} $$

According to the continuity of the soil, we know that $S\left( H \right)$ is the relative shear displacement between the anchor piers and the surrounding soil $S_{2}$, that is, $S\left( H \right) = \left( {S_{2} } \right)$.

### Calculation of the compression performance of the soil on the front of anchor piers based on semi-infinite body theory

When the anchor piers are deeply buried in the stable region, we can assume that the force of the soil around its front satisfies the condition for the distributed pressure in the finite area on a semi-infinite boundary plane. The lengths of the equivalent rectangle are $D_{e}$ and $L_{m}$, and there is uniform pressure with a degree of 1 on this equivalent area.

**Figure 11:**
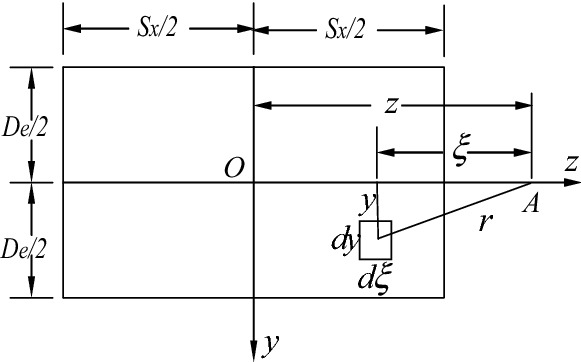
Analytical diagram of the compression area of the anchor piers.

In Fig. 11, the equivalent rectangle represents the range of loading. Now, we will solve the settlement $S_{ki}$ of a point $A$ on the symmetry axis of the equivalent rectangle, the distance of which to the center of the rectangle O is $z$. Therefore, this uniform unit force can be divided into several micro concentrated forces. Then, the contribution that every micro concentrated force makes to the settlement of point A and the effect of all the micro concentrated forces in the range of loading are determined. Finally, we obtain the actual settlement of point A at this time. Let $dP = 1/\left( {D_{e} L_{m} } \right)d\varepsilon dy$; the settlement of a point originated from this micro concentrated force can be calculated by Eq. (12)^28^, that is,

12 $$ dS_{ki} = \frac{{1 - \mu^{2} }}{\pi E} \cdot \frac{1}{{D_{e} L_{m} }}d\varepsilon dy \cdot \frac{1}{r} = \frac{{1 - \mu^{2} }}{\pi E} \cdot \frac{1}{{D_{e} L_{m} }} \cdot \frac{1}{{\sqrt {\varepsilon^{2} + y^{2} } }}d\varepsilon dy $$

Now, we only have to integrate $\varepsilon$ and $y$ on the whole loading plane to obtain the final settlement $S_{ki}$ of point $A$. Here, $r = \sqrt {\varepsilon^{2} + y^{2} }$.

13 $$ (S_{ki} ) = \frac{{1 - \mu^{2} }}{\pi E}\mathop \int \limits_{{Z - S_{x} /2}}^{{Z + S_{x} /2}} \mathop \int \limits_{{ - D_{e} /2}}^{{D_{e} /2}} \frac{1}{{\sqrt {\varepsilon^{2} + y^{2} } }}\frac{1}{{D_{e} L_{m} }}d\varepsilon dy = \frac{{1 - \mu^{2} }}{{\pi EL_{m} }}K_{ki} $$

where:

14 $$ \begin{aligned} & \left( {K_{ki} } \right) = \left( {\frac{{2\frac{z}{{L_{m} }} + 1}}{{\frac{{D_{e} }}{{L_{m} }}}}sh^{ - 1} \frac{{\frac{{D_{e} }}{{L_{m} }}}}{{2\frac{z}{{L_{m} }} + 1}} + sh^{ - 1} \frac{{2\frac{z}{{L_{m} }} + 1}}{{\frac{{D_{e} }}{{L_{m} }}}}} \right) \\ & \quad - \left( {\frac{{2\frac{z}{{L_{m} }} - 1}}{{\frac{{D_{e} }}{{L_{m} }}}}sh^{ - 1} \frac{{\frac{{D_{e} }}{{L_{m} }}}}{{2\frac{z}{{L_{m} }} - 1}} + sh^{ - 1} \frac{{2\frac{z}{{L_{m} }} - 1}}{{\frac{{D_{e} }}{{L_{m} }}}}} \right) \\ \end{aligned} $$

where $sh^{ - 1}$ is the inverse of a hyperbolic function.

If point $A$ is at the center point $O$ of the equivalent rectangle and $z = 0$, which is the location point of the stayed cable, the settlement is:

15 $$ \begin{aligned} & (S_{ki} ) = \frac{{1 - \mu^{2} }}{\pi E}\mathop \int \limits_{{ - S_{x} /2}}^{{S_{x} /2}} \mathop \int \limits_{{ - D_{e} /2}}^{{D_{e} /2}} \frac{1}{{\sqrt {\varepsilon^{2} + y^{2} } }}\frac{1}{{D_{e} L_{m} }}d\varepsilon dy \\ & \quad = \frac{{1 - \mu^{2} }}{{\pi EL_{m} }} \cdot \left( {\frac{{L_{m} }}{{D_{e} }}sh^{ - 1} \frac{{D_{e} }}{{L_{m} }} + sh^{ - 1} \frac{{L_{m} }}{{D_{e} }}} \right) \\ \end{aligned} $$

After determining the settlement $$ (S_{ki} )$$ of the center point of the equivalent rectangle under uniform unit pressure, we can obtain the compression performance of the soil on the front of anchor piers $$\left( {S_{3} } \right)$$ under the action of tension $$T$$, that is:

16 $$ (S_{3} ) = TS_{ki} = \frac{{T\left( {1 - \mu^{2} } \right)}}{{\pi EL_{m} }} \cdot \left( {\frac{{L_{m} }}{{D_{e} }}sh^{ - 1} \frac{{D_{e} }}{{L_{m} }} + sh^{ - 1} \frac{{L_{m} }}{{D_{e} }}} \right) $$

From the above analysis, the total displacement of the system is

17 $$ \left( S \right) = \left( {S_{1} } \right) + \left( {S_{2} } \right) + (S_{3} ) = \frac{{TL_{1} }}{{E_{l} A_{l} }} + \frac{T}{{aD_{e} L_{m} E_{e} }} \cdot \frac{{2exp\left( {aH} \right)}}{{exp\left( {2aH} \right) - 1}} + \frac{{T\left( {1 - \mu^{2} } \right)}}{{\pi EL_{m} }} \cdot \left( {\frac{{L_{m} }}{{D_{e} }}sh^{ - 1} \frac{{D_{e} }}{{L_{m} }} + sh^{ - 1} \frac{{L_{m} }}{{D_{e} }}} \right) $$

## Analysis of examples

### Design parameters

**Figure 12:**
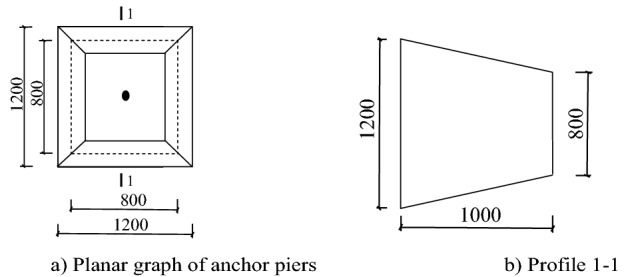
The parameters of anchor piers.

A new type of Debris-flow grille dam is proposed to be built with a height of 8 m. Column section 500 mm × 700 mm, spacing 5000 mm. The cross section of the beam is 400 mm × 300 mm, and the spacing is 4000 mm. The section steel adopts I-steel 45a, the spacing is 250 mm. The counterfort wall is 300 mm thick and 6500 mm high. Pile foundation adopts manual digging pile, pile by 1000 mm, 5000 mm deep. The concrete is C30; Stressed bar is HRB335; Stirrups is HRB300; Stay Cable is 3 $\emptyset$ s15.2. The design size of the anchor piers is shown in Fig. 12. In the Figure where $T = 2 \times 10^{5} N$; $L_{l} = 8500\;{\text{mm}}$; $E_{l} = 1.95 \times 10^{5} \;{\text{N/mm}}^{2}$; $A_{l} = 420\;{\text{mm}}$; $D_{e} = 1000\;{\text{mm}}$; $L_{m} = 1200\;{\text{mm}}$; $E_{e} = 3.0 \times 10^{4} \;{\text{N/mm}}^{2}$; $H = 1000\;{\text{mm}}$; $\mu = 0.2$; $E = 20\;{\text{N/mm}}^{2}$. The parameter of gully bed soil is shown in Table 1.

**Table 1:** The parameters of gully bed soil.

Elastic modulus (MPa)	Poisson's ratio	Cohesive strength (kPa)	Internal Friction angle (°)	Unit weight (kN/m^3^)
20	0.2	8.0	35.0	21

### Analysis of results

(1) The effect of the elastic modulus and Poisson's ratio of the surrounding soil on the displacement deformation of the anchor-pulling system.

The elastic modulus $E$ and Poisson's ratio $\mu$ are important parameters for calculating the displacement deformation of soil. They have something to do with both the properties of materials and the stress level. To analyze the effect of the physical parameter variation of the surrounding soil on the displacement deformation of the anchor-pulling system, we can study changing the elastic modulus and Poisson's ratio. The variation range of the elastic modulus is 15–45 N/mm^2^, and the variation range of Poisson's ratio is 0.15–0.25.

**Figure 13:**
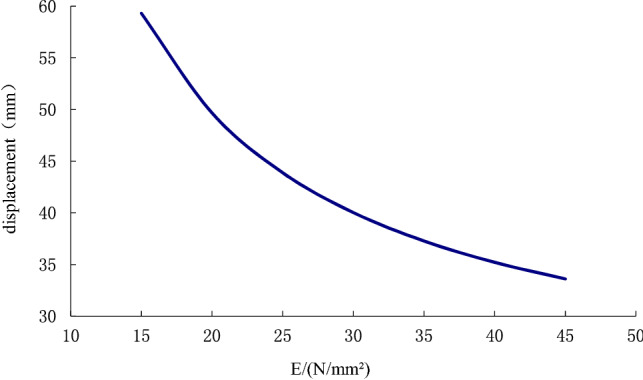
The effect of the elastic modulus *E*(15–45 N/mm^2^) of the surrounding soil on the displacement of the anchor-pulling system.

Figure 13 shows the variation curve in which the displacement deformation increases with the elastic modulus of the soil around the anchor pier. We can see that as the elastic modulus of the soil around the anchor pier increases, the displacement deformation decreases gradually. When the elastic modulus is in the range of 15–35 N/mm^2^, the curve is steep, and the decrease in deformation is apparent. After 35 N/mm^2^, the curve becomes smooth, and the decrease in deformation tends to be stable.

**Figure 14:**
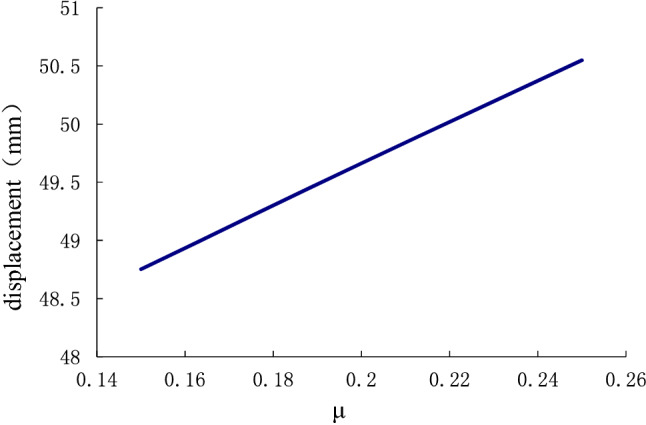
The effect of Poisson's ratio $\mu$(0.15–0.26) of the surrounding soil on the displacement of the anchor-pulling system.

**Figure 15:**
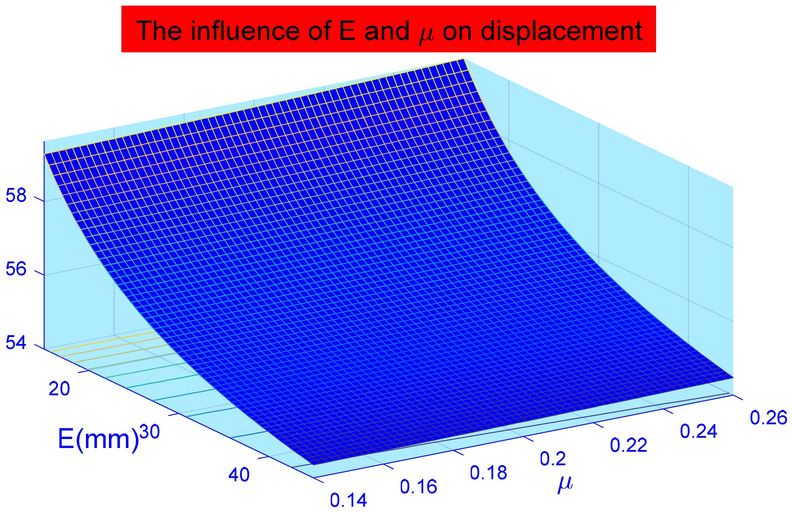
Influence of elastic modulus E (15–45 N/mm^2^) and Poisson's ratio $\mu \left( {0.15 - 0.26} \right)$ on deformation of anchor system.

In Fig. 14, the displacement deformation increases linearly with Poisson's ratio of the soil around the anchor pier. However, the total impact is not large. From calculation, the variation of elastic modulus of the soil around the anchor pier has nothing to do with elastic deformation of the stayed cable $$(S_{1} )$$, but mainly influences relative shear displacement between anchor piers and the surrounding soil $$(S_{2} )$$ and the compression performance of the soil on the front of anchor piers $$ (S_{3} )$$. where $$(S_{2} )$$ accounted for 89% and $$\left( {S_{3} } \right)$$ accounted for 11%. When the Poisson ratio increases, the displacement deformation also increases. Poisson's ratio has the greatest influence on the relative shear displacement $$(S_{2} )$$ of the anchor pier and soil, accounting for approximately 96.4%. The design parameters should be selected correctly during design. The influence of parameters on the deformation of anchor system is analyzed by using control variable method. The influence of a single variable on the results can be intuitively obtained. However, the elastic modulus E and Poisson ' s ratio $$\mu$$ of rock and soil are not independent. Therefore, Matlab is used to analyze the influence of the two aspects on the deformation of the tensile anchor system, and the results are shown in Fig. 15. It can be seen from Fig. 15 that the influence of elastic modulus E on the deformation of tensile anchor system is much greater than that of Poisson’s ratio $$\mu$$. And the variation of the curve is basically the same, so the interaction between the two is weak.

(2) The effect of the design parameters of anchor piers on the displacement deformation of the anchor-pulling system.

**Figure 16:**
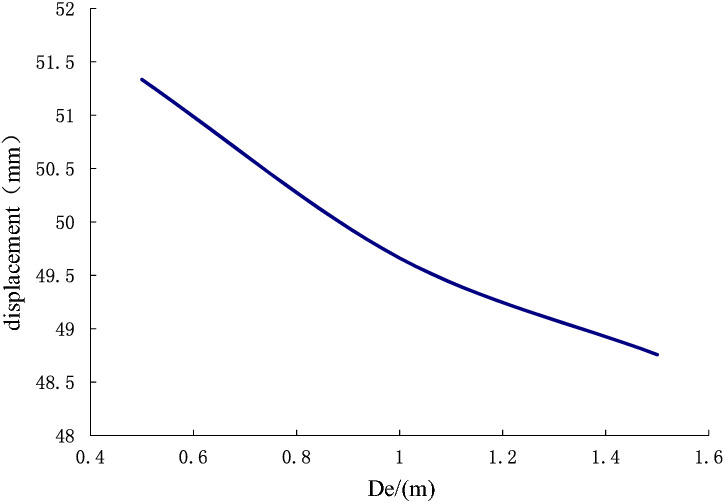
The effect of equivalent width $D_{e}$(500–1500 mm) on the displacement of the anchor-pulling system.

**Figure 17:**
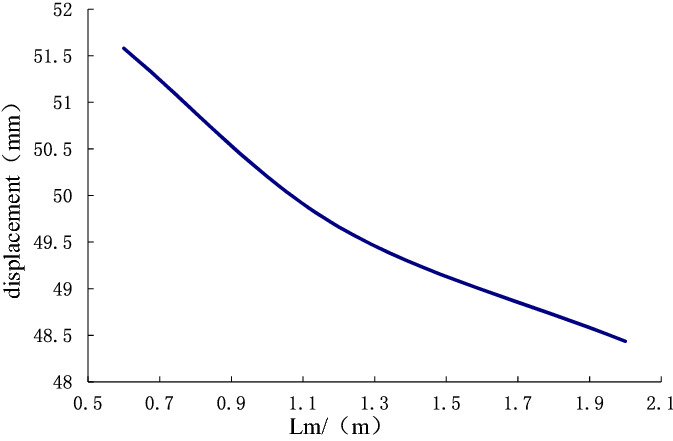
The effect of equivalent length $L_{m}$(600–2000 mm) on the displacement of the anchor-pulling system.

The design parameters of anchor piers include the equivalent width $D_{e}$, length $L_{m}$ and height $H$. Different design parameters have varying effects on the displacement deformation of the anchor-pulling system. Keep other parameters unchanged and let $D_{e}$ vary in 0.5–1.5 m, $L_{m}$ vary in 0.6–2.0 m, and $H$ vary in 0.5–1.5 m. Analyzing their effect on the displacement deformation of the anchor-pulling system, the results are shown in Figs. 16 and 17.

As illustrated in Figs. 16 and 17, the effects of the design parameters of the anchor piers on the displacement deformation of the anchor-pulling system are almost the same. As the size increases, the displacement deformation gradually decreases, and the front section decreases quickly, while the rear section becomes gradually smooth. Here, the equivalent width $D_{e}$ and length $L_{m}$ mainly affect the compression performance of the soil on the front of anchor piers $\left( {S_{3} } \right)$. The anchor piers can be seen as rigid bodies where horizontal displacement takes place. Increasing the size means increasing the contact area between the anchor pier and soil body. With this increase, the compression performance of the soil on the front of the anchor piers decreases. However, the effect of the height $H$ on the displacement deformation of the anchor-pulling system is the contribution to the relative shear displacement between the anchor piers and the surrounding soil $(S_{2} )$. When $H$ grows, $(S_{2} )$ grows accordingly. However, theoretically, the larger the effect of the size, the better it is. Because of the constraint of topographic conditions, construction conditions and economic benefits in practical engineering, it is necessary to choose the best size. the anchor pier provides enough anchor force and saves all kinds of resources. The best design dimensions suggested are $D_{e}$ = 1.2 m–1.8 m, $L_{m}$ = 1.5 m–2.5 m, and $H$ = 1.0 m–1.6 m.

**Figure 18:**
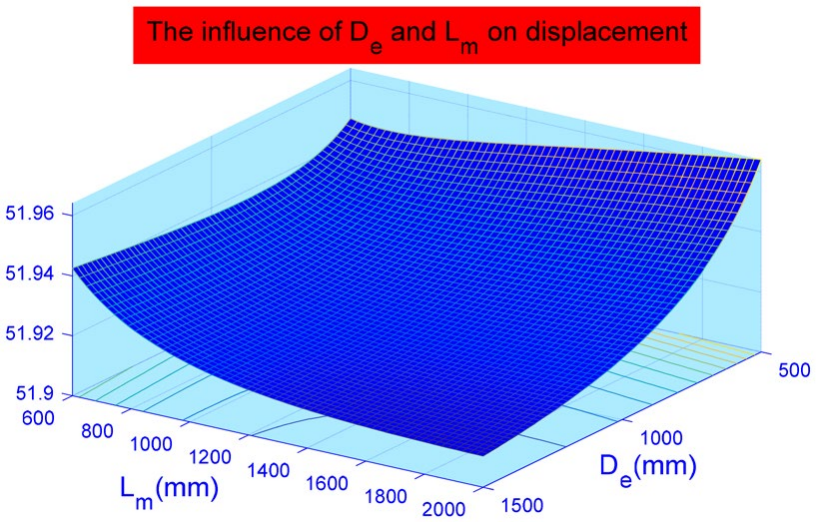
Influence of Anchor Pier Width $D_{e} \left( {500 - 1500\;{\text{mm}}} \right)$ and Anchor Pier Height $L_{m} \left( {600 - 2000\;{\text{mm}}} \right)$ on Deformation of Anchorage System.

It can be seen from Fig. 18 that the width $D_{e}$ and the height $L_{m}$ of anchor pier influence each other greatly. When $D_{e}$ is 600 mm, with the increase of $L_{m}$, the deformation of tension anchor system will first decrease and then increase. When $D_{e}$ is greater than 800 mm, with the increase of $L_{m}$, the deformation of tension anchor system will continue to decrease. And with the increase of $L_{m}$, the decreasing trend is more obvious. When $L_{m}$ is 500 mm, with the increase of the height of the anchor pier $D_{e}$, the deformation of the anchor system will increase first. When $L_{m}$ is greater than 800 mm, with the increase of $D_{e}$, the deformation of the anchor system will continue to decrease. But the decreasing trend is not much different.

### The numerical validation

#### The establishment of the finite element model

**Figure 19:**
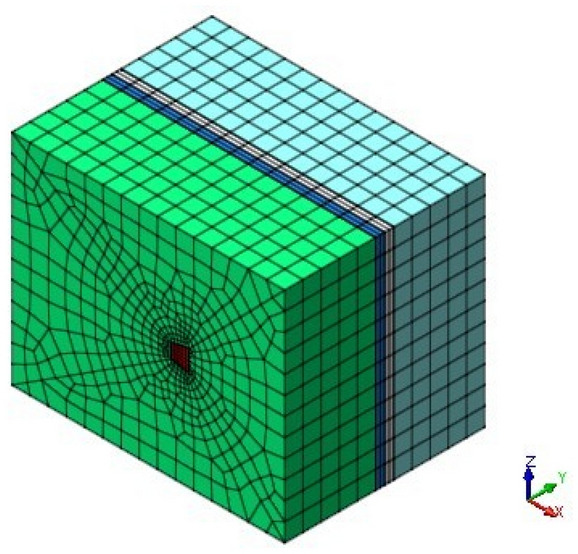
Finite element model of the anchor-pulling system and surrounding soil.

When the finite element model of the anchor-pulling system and surrounding soil is created, the constitutive model of the surrounding soil uses the Mohr–Coulomb elastoplastic model. The anchor pier and surrounding soil use eight nodes as oparametric elements, such as solid45, of which the basic grid unit is cubic units. When the grid is divided, the grid between the anchor pier and the surrounding soil contact is dense. The LINK10 unit is used to simulate cables, which have a bilinear stiffness matrix. It can simulate not only tensile bar units but also compressed bar units. For example, when the pull-up option is used alone, if the unit is under pressure, its stiffness disappears, so it can be used to simulate the relaxation of cables or chains. This feature is very significant for the static problem of wire rope, which uses a unit to simulate the entire cable. It can also be used for dynamic analysis with inertial or damping effects when the needed relaxation unit should pay attention to its performance rather than its movement. The soil is homogeneous. The soil physical parameters and structure design parameters are consistent with the theoretical calculation parameters mentioned above. The tensile force of the cable is exerted on the nodes as a force. The top surface of the model is free, and the normal displacements of the remaining faces are constrained such that the displacements are zero. The contact of the anchor pier and surrounding soils is a rigid-flexible surface-to-surface contact element to reflect the interaction. The surface of the anchor pier is regarded as the "target" surface, and the surface of the soil body is regarded as the "contact" surface. The coefficient of friction and normal penalty stiffness are 0.35 and 0.15, respectively. The scope of interaction between the anchor pier and the surrounding soil in the model is taken as 15 m × 11 m × 12 m, referring to past experience in engineering and the research data of the effect scope that the related anchors have had on the soil. The values of the model geometric parameters and physical and mechanical parameters are the same as in “Design parameters” section. The finite element model is shown in Fig. 19.

#### Research on finite element model grid

**Figure 20:**
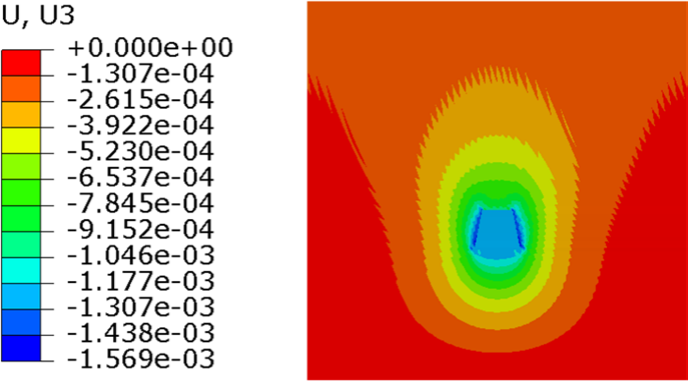
Condition 1 stress cloud diagram.

**Figure 21:**
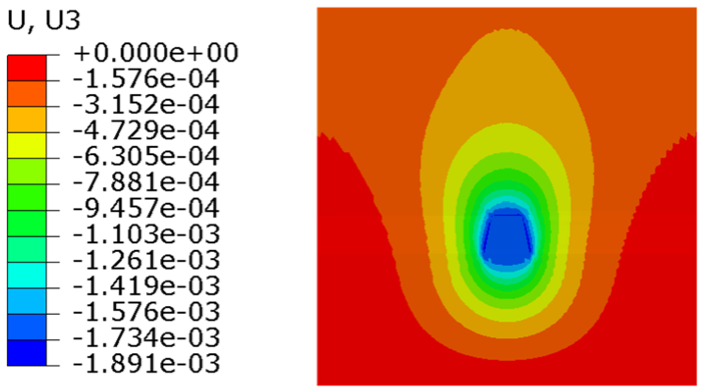
Condition 1 stress cloud diagram.

**Figure 22:**
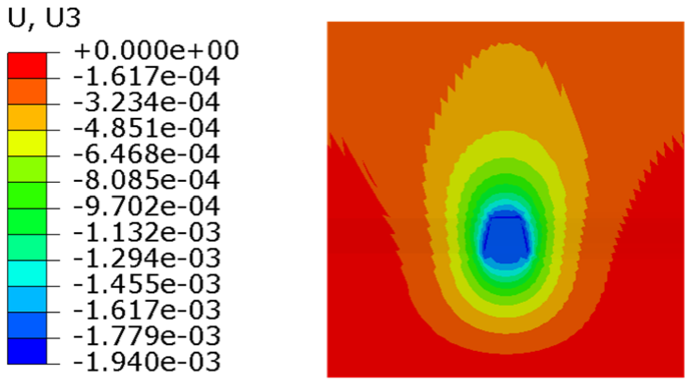
Condition 1 stress cloud diagram.

In order to verify the convergence of numerical simulation, the soil was divided into three different mesh sizes. Condition 1 is fine finite element meshing. The stress nephogram of condition 1 is shown in Fig. 20. Condition 2 is medium finite element mesh. The stress nephogram of condition 1 is shown in Fig. 21. Condition 3 is coarse finite element mesh. The stress nephogram of condition 1 is shown in Fig. 22. See Table 2 for specific grid division.

**Table 2:** Mesh size of three working conditions.

	Upper soil of anchor pier		Soil under anchor pier		Total iteration step
	Minimum size	Maximum size	Minimum size	Maximum size	
Mode 1	0.1	0.5	0.1	0.3	10
Mode 2	0.12	0.6	0.12	0.36	7
Mode 3	0.15	0.75	0.15	0.45	7

It can be seen from the stress nephogram of the three working conditions that the thicker the grid is, the greater the displacement of the anchor system is. The maximum displacement difference between condition 2 and condition 3 is 2.6%; the maximum displacement of condition 1 is 17% different from that of condition 2. The finer the mesh, the more accurate the numerical simulation results. But with the increase in computing time. It can be seen from Table 2 that the maximum iteration of condition 1 is 10 times, and the result will converge. The maximum iterations of condition 2 and 3 only need 7 times, and the results can converge.

#### The calculation results

**Figure 23:**
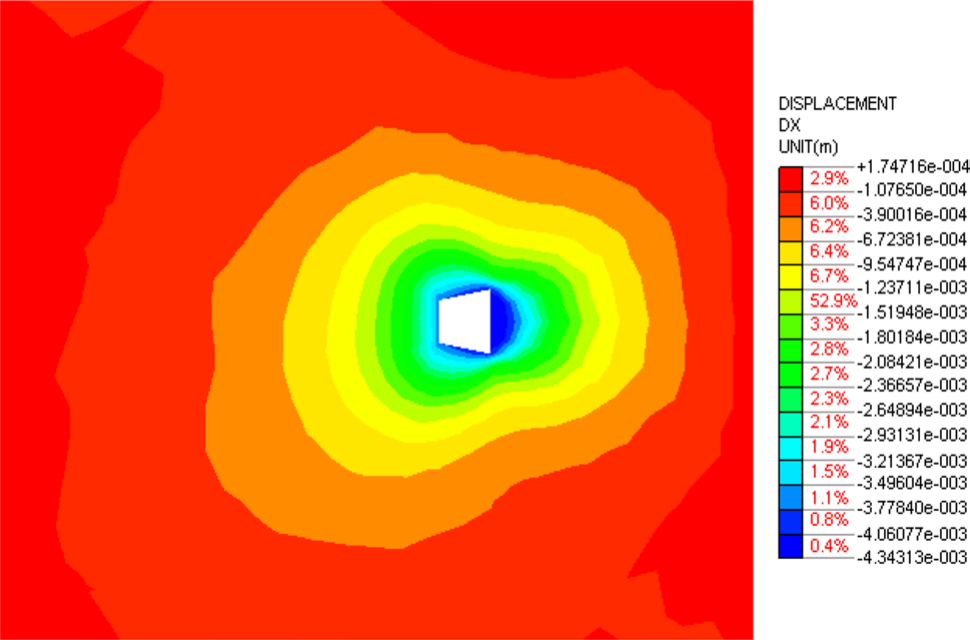
Displacement fringe of soil around the anchor piers for 100 kN.

**Figure 24:**
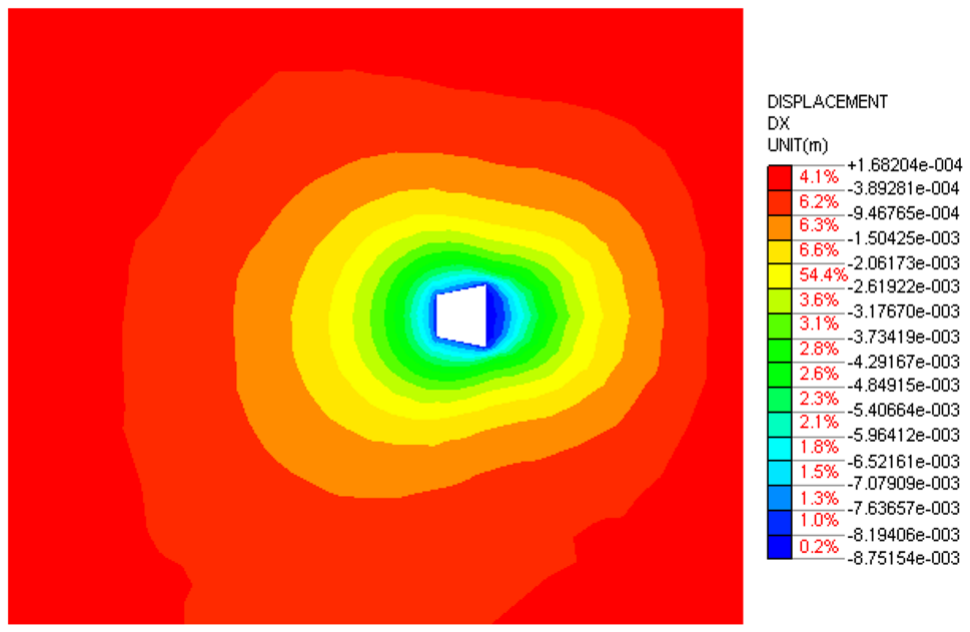
Displacement fringe of soil around the anchor piers for 400 kN.

Figure 23 and Fig. 24 are the displacement nephograms of the soil around the anchor piers for 100 kN and 400 kN, respectively. The soil displacement increases with increasing load, the affected area will increase and become uniform, and the area under load will also increase. The soil within the range of 1–3 m around the anchor pier is greatly affected, accounting for 80% of the total force. The soil around the anchor pier should be reinforced, and the anchoring force should be enhanced in the design.

**Figure 25:**
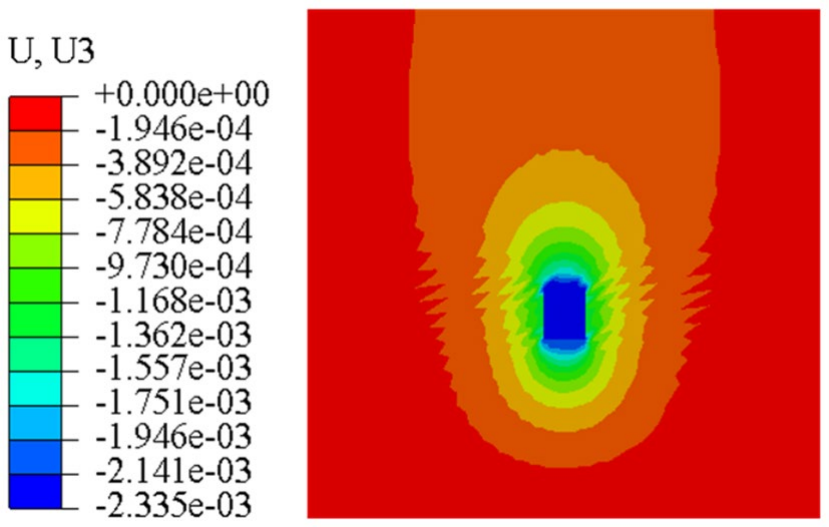
Top 800 mm, bottom 800 mm anchor pier stress nephogram.

**Figure 26:**
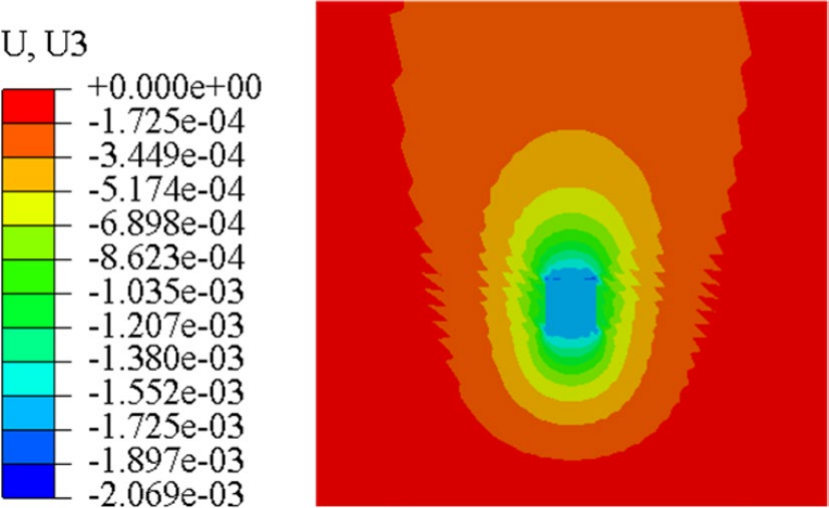
Top 1000 mm, bottom 1000 mm anchor pier stress nephogram.

**Figure 27:**
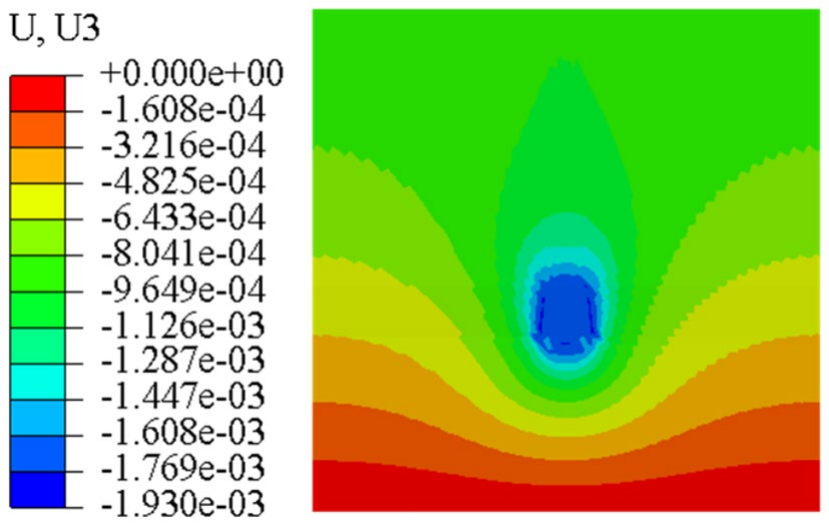
Top 800 mm, bottom 1000 mm anchor pier stress nephogram.

In order to further study the influence of anchorage pier size on the displacement and deformation of anchorage system, finite element models with different sizes are established by finite element method. The stress nephogram is shown in Figs. 25, 26 and 27.

From Figs. 25, 26 and 27, it can be seen that when the anchor pier is rectangular, the deformation of the tensile anchor system decreases with the increase of the size of the anchor pier, but the degree is small. When the anchor pier is trapezoidal, the material is small, but the deformation is more ideal than the rectangular. It can be seen that reasonable selection of anchor pier size is crucial, not blindly increase the size of anchor pier.

**Figure 28:**
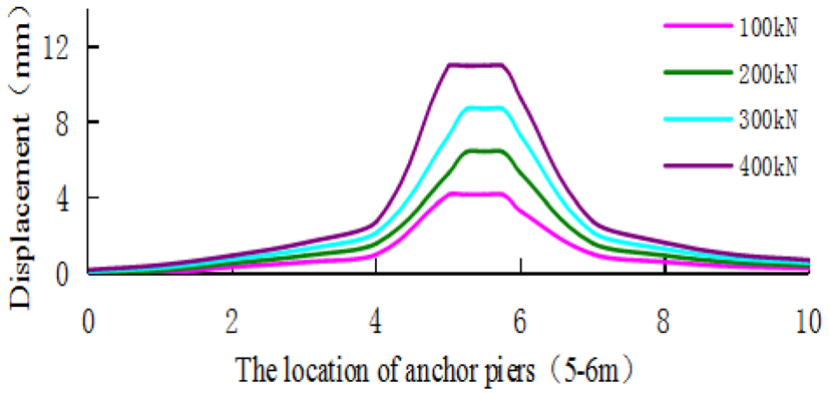
The displacement of soil around anchor pier.

**Figure 29:**
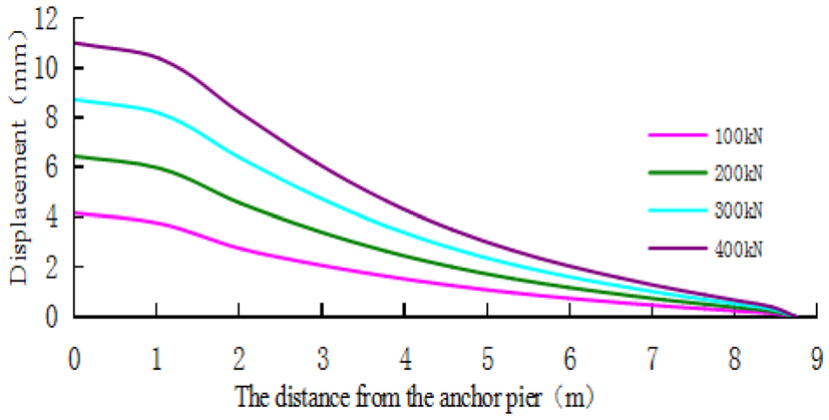
The horizontal displacement of soil along cable axis.

Figure 28 shows that the displacement of the soil around the anchor pier increases with increasing load, and the added value is obvious at approximately 2–3 mm. Figure 29 shows that the increase in load has a great effect on the soil in front of the anchor pier. As the load increases, the compressive deformation of the soil gradually increases. As the distance from the anchor pier increases, the displacement of the soil decreases, and the scope of influence gradually decreases. The displacement of the soil tends to be stable beyond 4–5 m from the anchor pier.

#### Comparison of theoretical calculation and numerical simulation results at the time of load variation

**Figure 30:**
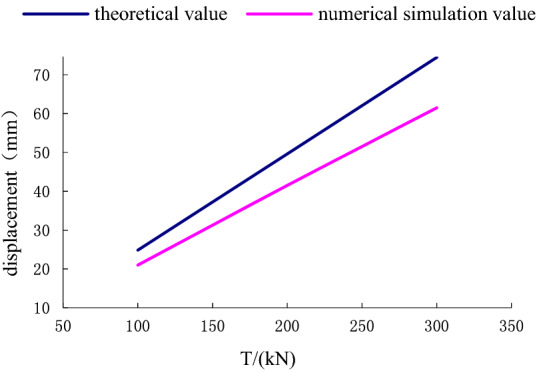
Comparison of theoretical calculation and numerical simulation results.

To verify the correctness of the theoretical calculation, we compare the theoretical calculation with numerical simulation results of displacement deformation of anchor-pulling system under different pulling force of stayed cable. The results are shown in Fig. 30, see Table 3 for data.

**Table 3:** Comparison between theoretical calculation and numerical simulation.

Pull (T/kN)	100	150	200	250	300
**Displacement and deformation (mm)**					
Theoretical calculation value	25	37	48	58	71
Numerical simulation value	21	30	39	48	59

As seen from Fig. 30, the theoretical and numerical simulation results are consistent, showing a linear growth trend. The slope difference of the two straight lines is approximately 5%, which meets the accuracy requirements of geotechnical engineering. As the restraint effect of the surrounding soil on the anchor pier is not fully considered, the theoretical calculation result is too large. The deformation of anchor $\left( {S_{1} } \right)$ in displacement deformation is the same, and the relative shear displacement $\left( {S_{2} } \right)$ of the anchor pier and the soil and the compressive deformation $(S_{3} )$ of the soil at the front end of the anchor pier are 1.25 times and 1.08 times the numerical simulation results, respectively. The change in $\left( {S_{2} } \right)$ in the calculation results is large and should be taken into account in the design.

## Conclusion

This paper provides a comprehensive analysis of the loading process and destruction mechanism of the anchor-pulling system for a new debris-flow grille dam, which was proposed by the author, establishes a calculation model of the anti-pulling force and displacement deformation in the anchor-pulling system, and systematically analyzes the influence of various parameters. The results can provide a theoretical reference for the promotion and application of the new debris-flow grille dam.

1. According to the destruction modes of the anchor-pulling system and the simplified calculation model, the formulas for calculating the anti-pulling force and the total displacement are given. The anti-pulling force $T_{b}$ is composed of two parts: the anti-pulling force $T_{1}$ provided by the frictional resistance of the sidewalls and the positive pressure $T_{2}$ on the front of the anchor piers. The total displacement of the system $\left( S \right)$ is composed of three parts: the elastic deformation $\left( {S_{1} } \right)$ of the stayed cable, relative shear displacement $(S_{2} )$ between anchor piers and the surrounding soil and the compression deformation $\left( {S_{3} } \right)$ of the soil on the front of anchor piers.
2. As the elastic modulus of the soil around the anchor pier increases, the displacement deformation decreases gradually. When the elastic modulus is less than 35 N/mm^2^, the curve is steep, and the deformation is obviously reduced. After more than 35 N/mm^2^, the curve gradually becomes gentle, and the displacement deformation tends to be stable. The change in elastic modulus mainly affects the relative shear displacement $\left( {S_{2} } \right)$ of the anchor pier and soil and the compressive deformation $\left( {S_{3} } \right)$ of the soil at the front end of the anchor pier. Among these, $(S_{2} )$ accounted for 89%, and $\left( {S_{3} } \right)$ accounted for 11%. When the Poisson ratio increases, the displacement deformation also increases. Poisson's ratio has the greatest influence on the relative shear displacement $(S_{2} )$ of the anchor pier and soil, accounting for approximately 96.4%. The design parameters should be selected correctly during design.
3. As the size of the anchor piers increases, the displacement deformation decreases gradually. Here, the equivalent width $D_{e}$ and length $L_{m}$ mainly affect the compression deformation $\left( {S_{3} } \right)$ of the soil on the front of anchor piers. The height $H$ mainly affects the relative shear displacement $\left( {S_{2} } \right)$. When $H$ grows, $(S_{2} )$ grows either. The constraint of topographic conditions, construction conditions and economic benefits should be considered in practical engineering. The suggested economic design dimensions are $D_{e}$ = 1.2 m ~ 1.8 m, $L_{m}$ = 1.5 m ~ 2.5 m, and $H$ = 1.0 m ~ 1.6 m.
4. Theoretical and numerical simulation results are consistent, showing a linear growth trend. The slope difference of the two straight lines is approximately 5%, which meets the accuracy requirements of geotechnical engineering. In the theoretical calculation results for displacement deformation, the relative shear displacement $(S_{2} )$ of the anchor pier and the soil and the compressive deformation $(S_{3} )$ of the soil at the front end of the anchor pier are 1.25 times and 1.08 times the numerical simulation results, respectively. The change in $(S_{2} )$ in the calculation results is large and should be taken into account in the design.
